# Cell-type and transcription factor specific enrichment of transcriptional cofactor motifs in ENCODE ChIP-seq data

**DOI:** 10.1186/1471-2164-14-S5-S2

**Published:** 2013-10-16

**Authors:** Chin Lui Goi, Peter Little, Chao Xie

**Affiliations:** 1 Department of Biochemistry, Yong Loo Lin School of Medicine, National University of Singapore, 8 Medical Drive, Singapore 117597, Singapore; 2 Life Sciences Institute, National University of Singapore, 28 Medical Drive, Singapore 117456, Singapore; 3 Singapore Centre on Environmental Life Sciences Engineering (SCELSE) Nanyang Technological University 60 Nanyang Drive, SBS-01N-27 Singapore 637551, Singapore

## Abstract

**Background:**

Cell type and TF specific interactions between Transcription Factors (TFs) and cofactors are essential for transcriptional regulation through recruitment of general transcription machinery to gene promoter regions and their identification heavily reliant on protein interaction assays.

**Results:**

Using TF targeted chromatin immunoprecipitation coupled with massively parallel sequencing (ChIP-seq) data from Encyclopedia of DNA Elements (ENCODE), we report cell type and TF specific TF-cofactor interactions captured *in vivo *through enrichments of non target cofactor binding site motifs within ChIP-seq peaks. We observe enrichments in both known and novel cofactor motifs.

**Conclusions:**

Given the regulatory implications which TF and cofactor interactions have on a cell's phenotype, their identification is necessary but challenging. Here we present the findings to our analyses surrounding the investigation of TF-cofactor interactions encoded within TF ChIP-seq peaks. Novel cofactor binding site enrichments observed provides valuable insight into TF and cell type specific interactions driving TF interactions.

## Background

Transcription Factors (TFs) are protein complexes responsible for the recruitment of basic transcription machinery to DNA [1]. At the molecular level, gene regulation is achieved via the binding of TFs to DNA but increases in complexity at a cellular level.

Distinct transcriptional programs made of interacting networks of TFs each regulating a subset of genes work cooperatively to generate the diversity of cells seen in multicellular organisms.

Mapping of TF interactions within such net- works are important for understanding of their control over gene expression and higher order functions such as cell fate determination [2]. Although cell type specific expression of TFs have been identified, it is through combinatorial binding with partner TFs that the recruitment and formation of preinitiation complexes and RNA polymerases required for the transcription of cell type specific genes is achieved. Identification of such unique combinatory TF binding patterns occurring in a cell type specific manner is necessary for understanding of the unique transcriptional programmes which givejk rise to the repertoire of cell types seen in a multicellular organism [1,3].

Cell type agnostic interactions do exist between TFs and are TF specific where master regulator TFs like the Signal Transducer and Activator of Transcription (STAT) bind with its cofactors to activate transcription of gene targets regardless of cell types [4,5].

While existing methods of discovering TF-cofactor interactions require protein interaction assays, such as protein complex immunoprecipitation, or two hybrid screens which are low throughput, costly and non-indicative of *in vivo *conditions [6-10].

Specificity of TFs to their cognate binding sites have been well studied and with ChIP-sequencing (ChIP-seq) for chromatin immunoprecipitation (ChIP) coupled to ultra-high throughput massively parallel sequencing [11]. During ChIP-seq, DNA binding proteins are treated with a fixative agent, usually formaldehyde, and cross-linked to their bound DNA before it is extracted and the chromatin sheared to a size of 100-300 bp [12,13]. The resulting protein(s) of interest in this case TFs are immunoenriched using an antibody precipitation targeted at the TF. Thereafter, the cross-links are reversed and the DNA purified and analysed by high-throughput DNA sequencing. Regions within the genome significantly mapped back onto are identified as potential protein-DNA interaction sites or peaks [11].

Sequenced regions include those bound by cofactors is largely due to the fixation step during ChIP where fixation not only occurs between the antibody targeted TFs and bound region but similarly with cofactors in a TFBS-TF-Cofactor-TFBS manner [14]. As a result, protein-DNA interaction sites sequenced are not exclusive to the targeted TFs but also of their cofactors. Although, this has generally been viewed as noise and an artefact of the ChIP method impeding discovery of canonical TFBS motifs belonging to the targeted TF, documentation and support of enrichment of cofactor bound regions have been reported ranging from areas concerning peak calling techniques to genome-wide binding studies [15,16].

Thus, identification and scanning of bound genomic regions by the ChIP targeted TFs and cofactors *in vivo *for transcription factor binding sites (TFBS) can be achieved and their co-occurrences used as a proxy for their interactions. While analysis of TF ChIP-seq peaks data is much more scalable for investigating far larger libraries of TFs.

Given the existence of cell type and TF specific TF-cofactor interactions as well as the challenges in conventional methods of TF-cofactor discovery, we set out to explore *in silico *alternatives to analysing Cell type and TF specific TF-cofactor interactions from TFBS motif enrichments within ChIP-seq peaks. For cell type specific enrichments, we screened peaks for enrichments in non-canonical motifs, motifs with no known associations with the immuno-targeted TFs, across mutliple cell types (> 10). Whereas for TF specific enrichments >20% again in non-canonical motifs in > 3 cell types targeting the same TF.

In our study we used human ChIP-seq data from The Encyclopedia of DNA Elements (ENCODE) Project [17]. In the June 2011 release by ENCODE, the Encode Transcription Factor Super Regulation Track integrates previously separate tracks containing ChIP-seq datasets from 81 experiments onto a single dataset which is mapped onto the latest human genome assembly (GRCh37/hg19). The release includes ChIP-seq experiments belonging to a variety of TFs carried out using different cell types.

In contrast to previous reports of cofactor sig-natures within TF ChIP-seq peak data [18] for our analysis, to our knowledge is the largest, spanning 81 ChIP-seq datasets after filtering.

For this study, we aim to identify TF-cofactor interaction networks through careful screening and analysis of transcription cofactor motifs captured by TF ChIP-seq as well as uncover nuances in their interaction specificities relating to cell types, and individual TFs.

## Results and discussion

In the following, we begin with an overview of analyses conducted on the ChIP-seq dataset as well as report significant co-occurring TFBS motifs belonging to both validated and predicted cofactors. Of these, some exhibit *Cell type *as well as *TF *specificity upon applying criteria specific filters.

### Overview

Using ENCODE's recent release, a total of 425 ChIP-seq experiments studying 122 TFs in 95 different cell cultures were considered initially for this study totaling 1,702,787 unique ChIP-seq peaks.

We removed experiments investigating basic transcription machinery Polymerase I, II and III and non-canonical TF CTCF. Peaks belonging to high occupancy regions, that is being ubiquitous across ChIP-seq experiments regardless of conditions were also not considered. Finally, TFs investigated in only a single cell type as well as those without matched DNA binding site motifs were also removed. Also excluded from analysis were peaks showing extensive overlaps with peaks of other TF ChIP-seq experiments targeting different TFs (67,246 out of 1,702,787). Accumulation of functionally unrelated DNA binding factors in regions known as 'High-Occupancy Target' (HOT) regions have been documented [19]. Nucleation at these sites has been shown mainly to be a result of protein-protein interactions [19-21] while protein-DNA interactions if any are still unclear hence, excluded.

The resulting dataset containing 1,022,885 peaks from 81 unique ChIP-seq experiments across 46 unique cell cultures of various tissue types was chosen. 28 unique TFs remained after curation with a total of 56 mapped canonical TFBS motif position weight matrix (PWMs).

Finally, we looked for cell type specific as well as TF specific co-occurrences and identified a total of 134 such motifs (Tables 1 and 2). Examples of the above will be discussed in the following. All identified co-occurring motifs and potential factors are provided in the supplementary (Additional Files 1 and 2).

**Table 1:** Cell type specific co-occurring cofactor motifs.

Cell type	Co-occurring motifs (Jaspar and Uniprobe Motif ID)	Total
H1-hESC	MA0105.1, MA0145.1, MA0364.1, MA0375.1, MA0395.1, Zic1 secondary, Zic2 secondary, MA0154.1, MA0355.1, MA0364.1, Tcfap2b primary, Zic3 secondary	12
HeLa-S3	MA0145.1, MA0205.1, MA0375.1, MA0395.1, Zic2 secondary, Jundm2 secondary, MA0099.1, MA0272.1, MA0303.1	9
HepG2	MA0114.1, MA0017.1, MA0114.1	3
K562	MA0375.1, MA0395.1	2

Listed in the table are motifs found consistently enriched within ChIP-seq peaks investigating a particular cell type independent of the TF targeted by the ChIP process.

**Table 2:** TF specific co-occurring cofactor motifs.

Target TF	Co-occurring motifs (Jaspar and Uniprobe Motif ID)	Total
c-Fos	MA0419.1, MA0316.1, MA0314.1, MA0315.1, MA0060.1	5

c-Jun	MA0419.1, MA0018.2, Atf1 primary, Jundm2 primary	4

c-Myc	Sp4 secondary, Zfp161 secondary, MA0324.1, Tcfap2e primary, MA0112.1, Plagl1 primary, MA0374.1, MA0014.1, Tcfap2a secondary, Zic2 primary, Zic3 primary, Zic1 primary, MA0395.1	13

Egr-1	Sp4 secondary, Zfp161 secondary, MA0324.1, MA0375.1, Tcfap2e primary, MA0374.1, MA0014.1, MA0268.1	8

GABP	Sp4 secondary, Zfp161 secondary, MA0337.1, MA0324.1, MA0375.1, Tcfap2e primary, MA0048.1, MA0112.1, MA0374.1, MA0014.1, MA0145.1, MA0138.2, Tcfap2a secondary, Zic2 primary, Zic3 primary, Zic1 primary, MA0145.1, Zic2 primary, Tcfap2b primary	19

GATA-1	Mtf1 secondary, Tcfap2e secondary, MA0048.1, Srf secondary, Zfp105 primary, MA0402.1, MA0205.1, Sox13 secondary, Zic2 secondary, Zic1 secondary, Tcfap2a secondary, MA0154.1, Zic3 secondary, MA0057.1	14

GATA-2	Gata6 primary, Gata3 primary	2

MafK	MA0419.1, Mtf1 secondary, Tcfap2e secondary, Srf secondary, Zfp105 primary, MA0099.2, MA0150.1,	9

Max	Plagl1 primary	1

NFKB	MA0364.1, Sox13 secondary, MA0154.1, Zic1 primary	4

SP1	Sp4 secondary, Zfp161 secondary, MA0316.1, MA0315.1, Egr1 secondary, MA0112.1, Plagl1 primary, MA0374.1, MA0014.1, Sox13 secondary, Tcfap2a secondary, 19 MA0314.1, MA0060.1, MA0057.1, MA0395.1, MA0060.1	19

STAT1	Sox13 secondary	1

STAT3	MA0099.1, Jundm2 secondary, MA0272.1, MA0099.2, MA0303.1	5

USF-1	Plagl1 primary, MA0314.1, MA0060.1	3

YY1	Zic2 primary	1


Listed in the table are motifs found consistently enriched within ChIP-seq peaks investigating a particular TF independent of the cell type investigated.

### Proximally and distally located co-occurring motifs

**Figure 1:**
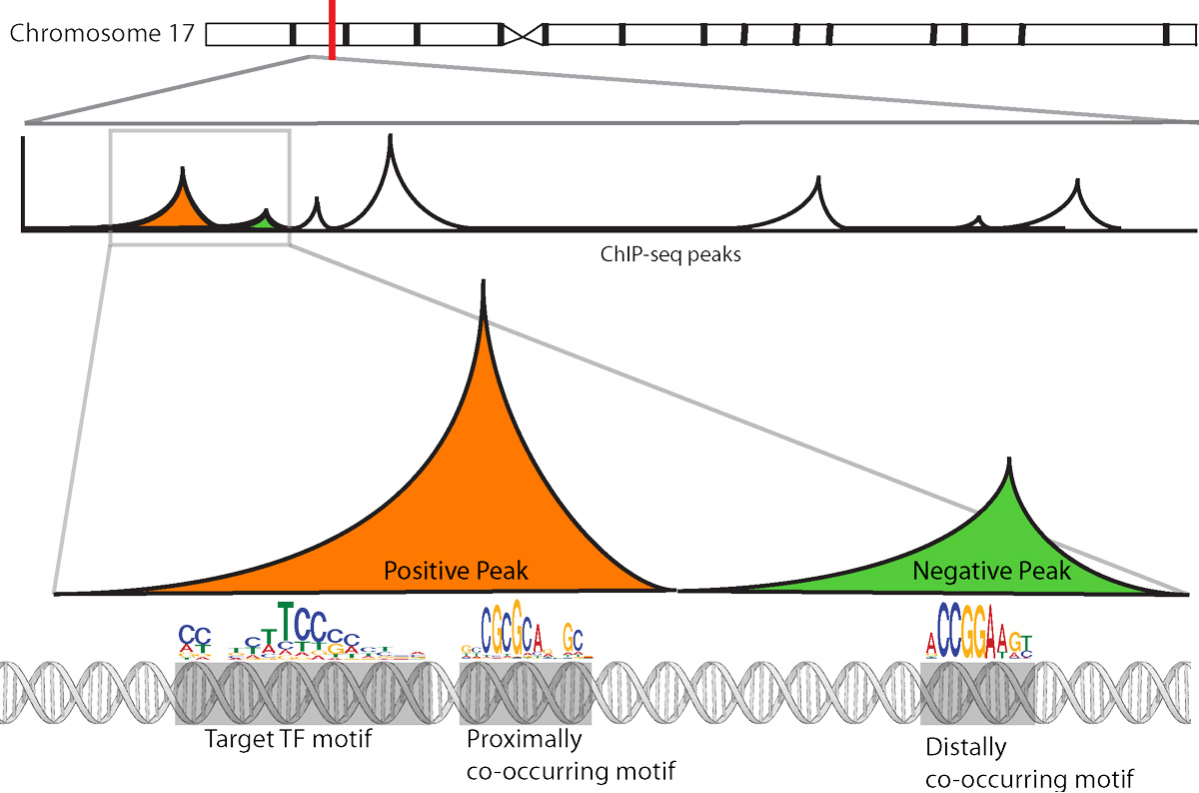
**Diagrammatic representation of proximally and distally co-occurring motifs**. In orange is a positive peak enriched with the target TF's motif as well as other proximally co-occurring motifs(s). In green, a negative peak absent for target TF's TFBS but enriched with cofactor motifs.

Peaks were later classified based on presence of canonical TFBS motifs belonging to the ChIP targeted TF (322,085 present and 700,800 absent) and a total of 75,955 non-canonical motifs were identified. Co-occurring motifs identified within peaks present and absent for the targeted TF's canonical motifs are thus classified as proximal and distal (Figure 1).

### Co-occurrence specificity of motifs

To identify TF-cofactor networks operating proximally and distally through cis- and trans-acting elements with respect to the ChIP targeted TFs, we searched for enrichments in co-occurring TFBS motifs within ChIP-seq peaks. We based our search on three parameters namely: (1) motif abundance; the enrichment of the co-occurring motif in the ChIP-seq peaks, (2) motif ubiquity; the presence of the co-occurring motif across peaks from different TF ChIP-seq experiments and the (3) uniqueness or dis-similarity the targeted TF's canonical motif(s) using similarity scores with *p*-values < 0.05. Potentially novel as well as known TF-cofactor pairs have been identified and selected examples will be discussed in the following. For the complete list of co-occurring motifs identified please refer to the supplementary tables provided (Additional files 1 and 2).

### Hepatocyte specific TF: HNF4α

**Figure 2:**
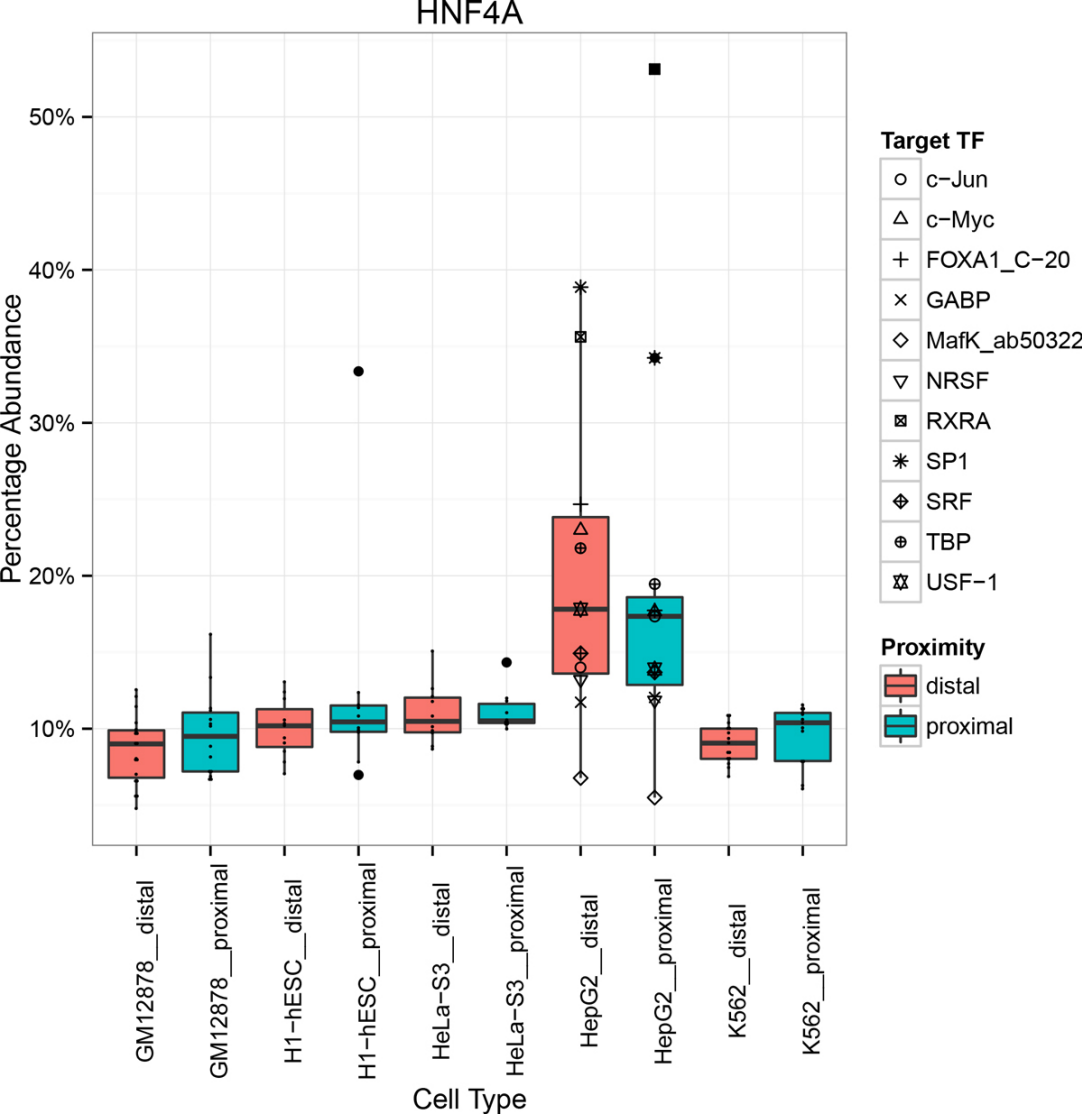
**Distribution of cell type specific cofactor HNF4A binding site motif across cell types**. Co-occurrence of transcription factor HNF4A's canonical motif in ChIP-seq peaks distal (pink) and proximal (green) with canonical motifs of ChIP targeted TFs across cell types. HNF4A binding site motif was found enriched in ChIP-seq peaks across experiments using HepG2 cell type. Dots represent individual ChIP-seq experiments.

The most striking cell type specific enrichment observed belonged to the motif of Hepatocyte nuclear Factor 4 alpha (HNF4α) [22]. The motif was found to be enriched both proximally and distally from ChIP targeted TF motifs found within the HepG2 cell lines regardless of the ChIP targeted TFs but not in other cell types (Figure 2).

HNF4α belongs to the superfamily of nuclear receptors known to be expressed endogenously in adult liver cell lines. Functionally, HNF4α is a ligand-dependent transcription factor which is a master regulator for liver-specific gene expression and forms homodimers as well as heterodimers with other TFs via its AF-2 transactivation domain [23].

Co-occurring with HNF4α motifs are the canonical motifs of 11 cofactor TFs (c-Jun, c-Myc, FOXA1, GABP, MafK, NRSF, RXRA, SP1, SRF, TBP, USF-1) both proximally and distally located peaks.

More cell type specific co-occuring TF motifs can be found in Additional file 3.

### TF specific co-occurring motifs

As in the earlier section, we selected co-occurring motifs fulfilling the specific criteria of motif enrichment (>20%) across > 3 cell types targeting the same TF.

108 TF specific motifs were identified with the majority (~ 83%) proximal to target TFBS motifs. Of these are experimentally verified co-factors of the target TFs as well as those whose identity as a co-factor has not been experimentally verified (see Additional file 4).

In the following we will discuss briefly 4 examples of such motifs showing TF specific enrichment (2 belonging to known cofactors and 2 potentially novel cofactors).

### Examples of known associations

#### Signal Transducer and Activator of Transcription 3 and Activator Protein-1

**Figure 3:**
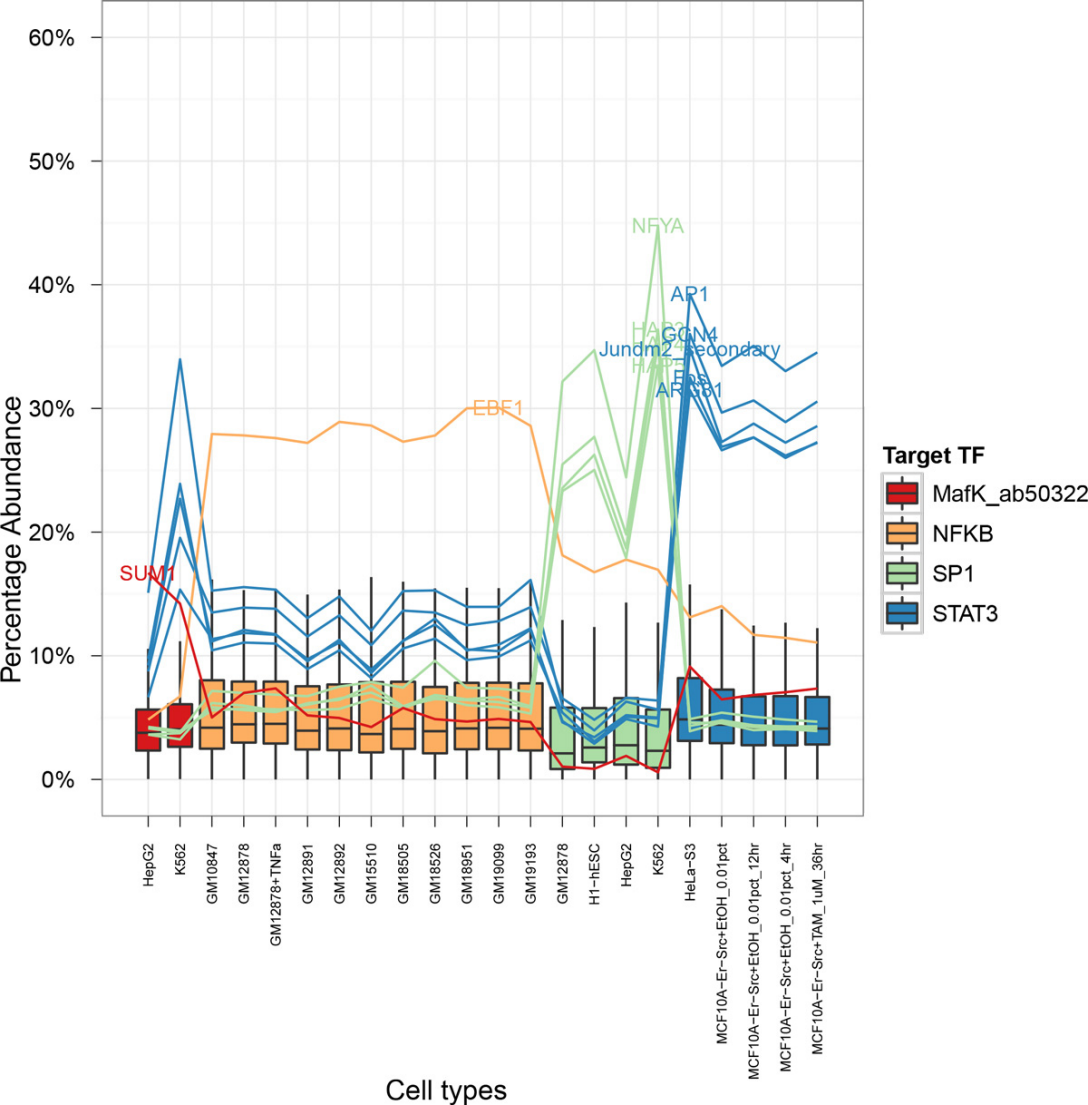
**TF specific co-occurring cofactor motifs**. TF specific co-occurring motifs identified upon applying the TF specificity screen. Box plot showing distribution of co-occurrence of motifs in ChIP-seq experiments investigating four TFs across various cell types. Overlaid on top of this is a line plot connecting TF specific motifs.

Upon applying the screening process, five TFBS motifs where found to be enriched proximally with canonical TFBS motifs of the Signal Transducer and Activator of Transcription Three (STAT3) (Jaspar motif ID: MA0144.1) regardless of cell type but not so in experiments targeting other TFs (Figure 3). Four out of five of these belonged to known cofactors and homologues of STAT3.

Three of the motifs identified were found to be canonical motifs of known STAT3 cofactor, Activator Protein 1 (AP-1) (Jaspar motif ID: MA0099.2) and its subunits c-Fos and c-Jun (Jaspar motif ID:MA0099.1 and Uniprobe motif ID: UP00103 secondary). Associations between STAT3 and AP-1 complexes are well characterised and their binding site motifs known to co-occur proximally together [24,25]. In addition, numerous assays con-ducted confirms their interactions both *in vitro *and *in vivo *[26-29].

AP-1 and STAT3 are known to be responsible for regulating the expression of genes mediating tissue repair and regeneration ubiquitously across cell types. The co-occurrences in the two's TFBS motifs in a cell type independent manner therefore is not surprising.

Of the remaining two motifs, one is a Saccharomyces cerevisiae homologue of the AP-1 sub-unit c-Jun, GCN4 (Jaspar motif ID: MA0303.1) which binds to the AP-1 specific sequences (*p*-value: 1.15405e-15) [30,31]. The other is a yeast TF responsible for regulating arginine-responsive genes [32-34].

Specificity Protein 1 (SP1) In a separate example of a TF specific co-occurrence of TFBS motifs, we observed the enrichment of 'CCAAT' family of TFs namely NFY, and the *Saccharomyces cerevisiae *homologues HAP3, HAP4 and HAP5 (Jaspar Motif ID: MA0060.1, MA00314.1, MA00315.1 and MA00316.1) in proximal peaks of ChIP-seq experiments targeting SP1 across cell types (Figure 3). It has been documented that SP1 and NFY share large overlaps in promoter occupancies across numerous genes [35-37] as well as functional assays testing for co-operativity between the two [38].

The positive identification of AP-1 and its subunits' motifs in ChIP-seq peaks studying to STAT3 but not in peaks studying SP1 and vis versa acts simultaneously as a positive internal control as well as a negative internal control for this study.

### Example novel cofactors

#### NFκB and EBF1

TF specific motif co-occurrences identified in our analysis which have not been experimentally validated to our knowledge previously as a cofactors belong to EBF1 (Jaspar motif ID: MA0154.1). The co-occurrence was observed in the proximal peaks of ChIP-seq targeting NFκB (Jaspar motif ID: MA0105.1) and the enrichment is fairly uniform across lymphocytes, embryonic stem cells, hepatocytes and human leukemia cells as shown in Figure 3.

EBF1 has been found to be important in the regulation of genes responsible for the normal progression of B cell development. Similarly, NFκB too has been shown to be essential for proper B cell development [39,40]. Hence, the possibility of the two participating in some form of co-operative binding to regulate B cell development genes is high.

#### Plagl1 and c-Myc

The motif of Plagl1 (Uniprobe motif ID: UP00088) was found enriched within peaks from ChIP-seq experiments targeting c-Myc across cervix adenocarcinoma cells (HeLa), human leukemia cells (K562), hepatocytes (HepG2), human breast adenocarcinoma cells (Mcf-7), lymophocytes (GM12878) and promyelocytic cells (NB4).

**Figure 4:**
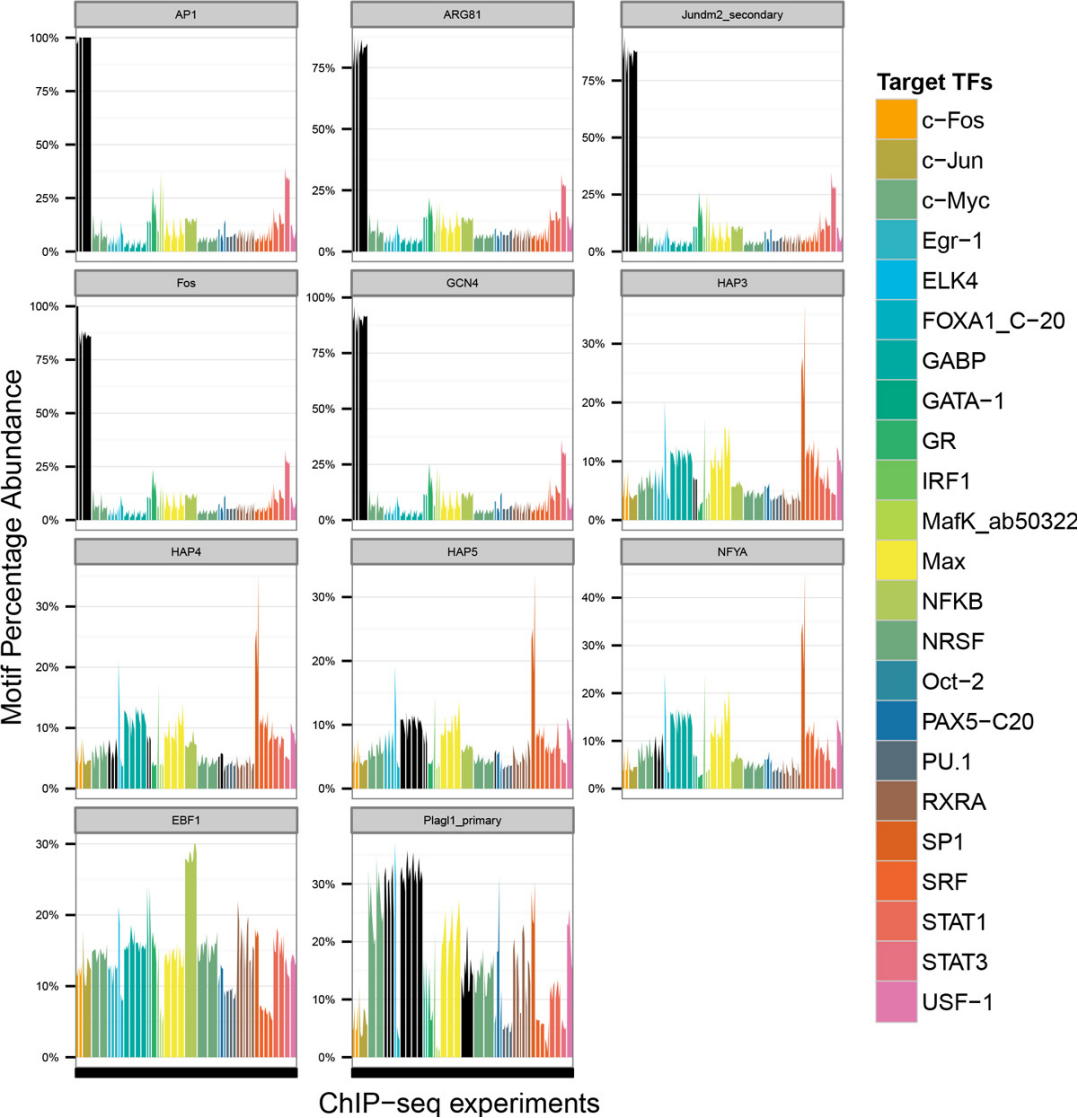
**Proximal co-occurrence of motifs with target TF canonical motifs**. Significant TF specific co-occurrences of TF motifs with ChIP targeted TF canonical motifs in ChIP-seq peaks. Shown on the x-axis are the individual ChIP-seq experiments and presence of selected motifs in ChIP-seq peaks of these experiments is reflected on the y-axis. In black are ChIP-seq experiments targeting TFs which show high similarity with the motif in question. Colors represent the TF targeted for the ChIP-seq experiment. Known associations identified include the motifs belonging to AP1, ARG81, Jundm2 secondary, Fos and GCN4 which were enriched in peaks where STAT3 was the target TF. Similarly those of HAP3, 4, 5 and NYFA were found enriched in peaks where SP1 was the target TF. Examples of novel TF specific enrichments include EBF1 with NFKB and Plagl1 with c-Myc.

Plagl1 and c-Myc are known regulators of the cell cycle and Plagl1 have been associated with inducing cell cycle arrest and apoptosis [41] while c-Myc involved in cell proliferation and apoptosis [42]. It is still unclear if the two TFs are true cofactors and will be a potential target for verification experimentally. In addition, the motif of Plagl1 was also identified in the peaks targeting SP1 and it is known that Plagl1 binds with SP1 response elements [43,44]. A summary figure showing the enrichment of the above mentioned pairing can be found in Figure 4.

## Conclusions

Our analyses have uncovered enrichments of known and novel TF cofactors combinations occurring in cell-type and TF specific manner worth investigating. Through the examples discussed we've shown the retrieval of 3 verified cofactors including HNF4A in hepatocytes, STAT3 and AP-1, and NFY-A and SP1 as well as novel co-occurrences such as EBF1 and NFKB suggesting the possibility of the two being cofactors.

Thus, it is apparent following critical examination of enrichments in non-canonical TFBS motifs in ChIP-seq data that cofactor motifs signatures are indeed detectable and retrievable through rigorous screening as described in our study.

In conclusion, we have shown through careful examination of motif enrichment in ChIP-seq data that not only are global cofactors of TFs be identified but also criteria specific binding partners. This could potentially be used for quick identification of potential cofactors of newly characterised TFs not only in humans but also other model organisms.

Such analyses as described in our study will prove be invaluable as more TFs are interrogated using ChIP-seq as the cost of next generation sequencing continues to become more affordable.

## Methods

To identify potential cofactor TFBS motifs from ChIP-seq data, we began with the collection of TF ChIP-seq experimental data as well as a representative list of known TFBS motifs. Next, we applied a series of procedures to process and screen for significant motifs exhibiting any of the two qualities: Cell type and TF specificity.

### Preparation and integration of data

#### TF ChIP-seq data

In our analysis, we used TF ChIP-seq experimental data retrieved from the Integrated Transcription Factor Track in the Data Coordination Center of the ENCODE project [45,46]. The Integrated Transcription Factor Track was downloaded as a flat data matrix consisting of the genomic coordinates of ChIP-seq peaks corresponding to cell type of the experiment and TF investigated. Data from a total of 425 ChIP-seq experiments were retrieved, featuring 122 TFs targeted using a total of 148 TF specific antibodies in 95 different cell cultures be-longing to 71 unique cell types some treated with biological or non-biological agents.

All 1,702,787 peaks were mapped to their appropriate DNA sequences belonging to the latest human genome assembly (GRCh37/hg19) [45,46] using the getSeq() function from the *BSgenome.Hsapiens.UCSC.hg19 *package in R [47].

#### TFBS motif Position Weight Matrices (PWMs)

Identification of co-occurring TFBS motifs using motif finding algorithms requires consensus Position Weight Matrices (PWMs) which summarise DNA profiles of DNA sites bound by the DNA binding domain (DBDs) of a TF. We retrieved curated PWMs from two leading open-access TFBS motif databases: JASPAR CORE 2009 and UniPROBE Mouse [48,49]. Entries from the two databases show little overlap, representative of all known TFBS motifs used to search TFBS motifs in ChIP-seq peaks.

#### Matching target TFs to PWMs

A curated list of Tar-get TF canonical TFBS PWM(s) was retrieved from Ensembl [50].

#### A high level procedure for selecting ChIP-seq peaks and scanning for enriched cofactor TFBS motifs

We begin by examining data from each ChIP-seq experiment based on the following: the *nature *of the targeted TF, the *number *of experiments targeting the same TF*, peak density *of genomic regions and associated peaks, and the *availability *of target TF TFBS motif PWM(s).

Thereafter, we searched for proximally and dis-tally located co-occurring motifs by scanning for motifs in peaks positive for the target TFBS motif and peaks negative for the target TFBS motif. Motifs identified in the former represent motifs found in close proximity with the target TFBS while the motifs identified in the later represent motifs located distally from the target TFBS.

Finally, we determined significant and non-ubiquitous co-occurrences and screened them for: *Cell type *and *TF specificity*.

### Data curation

#### Nature of ChIP-seq targeted transcription factors

For meaningful analysis of TF-cofactor interactions, ChIP-seq experiments targeting general transcription machinery such as Polymerase II, III and the TATA-binding protein (TBP) were not considered for analysis. Similarly, the *non-*canonical TF such as CTCF were also removed.

Justifications for considering CTCF as a non-canonical TF Initially considered as a canonical TF, CCCTC-binding Factor (CTCF) shows similar genomic distributions with TFs such as STAT1 and NRSF [51]. However, CTCF has also been shown to exhibit additional non-canonical qualities acting as a transcriptional insulator as well as binding with multiple divergent DNA motifs [52]. In addition, CTCF exhibits large deviations in its genome-wide distribution from Transcription Start Sites (TSS) when compared to general transcription machinery and only ≈ 20% of its binding sites show promoter-proximal localisation [53]. Considering CTCF's non-canonical TF qualities, experiments targeting CTCF were therefore not included for analysis.

#### Number of cell type specific experiments targeting the same TF

'Orphan' ChIP-seq experiments without 'sister' experiments investigating the same TF but in different cell types were not selected for further analysis as we were unable to ascertain occurrence specificity of the co-occurring motifs.

#### Peak occupancy in mapped regions

HOT and COLD regions Individual peaks from each ChIP-seq experiment were curated based on the TF occupancy of the regions they are found in. Regions observing significant overlaps in peaks from multiple TFs (ChIP-seq experiments), henceforth referred to as High-Occupancy-Target (HOT) regions, are known to se-quester DNA binding factors but yet not much is known about their formation. As we were unable to determine if the motifs in the DNA sequences co-immunoprecipitated were truly bound by a cofactor or simply by another factors in the larger protein aggregates we chose not to include the peaks coming from these HOT regions into our analysis.

The arbitrary cutoff set to delimit such HOT regions requires the overlapping of peaks from more than 50% of all ChIP-seq experiments investigated. Peaks found in these HOT regions will be henceforth referred to as "HOT peaks" and the rest as "COLD peaks" for nomenclatural reasons. Availability of Target TF TFBS motif PWM(s) Experiments targeting TFs without any matched TFBS motif PWM(s) from the curated list of TF PWMs retrieved from Ensembl earlier were removed from analysis.

TFs which were matched to multiple canonical motifs were also observed due to multiple DBDs or have DBDs with alternative conformational states.

#### Proximally and distally co-occurring motifs

As shown in Figure 1, there exists proximally and distally located co-occurring motifs captured by ChIP-seq. Using canonical TFBS motif(s) for each TF, we scanned the corresponding ChIP-seq peaks for their presence and segregated the peaks into two; positive or negative. Next, we scanned both positive and negative peaks for TFBS motifs from our com-piled library of TFBS motifs.

Enrichment of TFBS motifs excluding that of the target TF's in positive peaks were considered to be proximal co-occurrences where the both target TF motif and enriched motif share the same ChIP-seq peak. Motifs enriched in peaks negative for the target TF's motif(s) were grouped as distally co-occurring. See Figure 1 for a diagrammatic representation of positive and negative peaks as well as proximally and distally located co-occurring motifs.

### Motif enrichment abundance

Abundance scores for each of the identified co-occurring motifs were assigned based on the motif's presence across the ChIP-seq peaks investigated regardless of its enrichment within each peak.

#### Ubiquity of motifs across experiments

Some motifs were observed to co-occur in ChIP-seq peaks both abundantly within a ChIP-seq experiment as well as ubiquitously across ChIP-seq experiments regardless of the cell type or the targeted TF. Such *non-*specific motifs were discarded from further analysis as we proceeded to screen for various criteria specific co-occurrences of TFBS motifs in the second part of our analyse.

This was achieved using two arbitrary thresholds, such that the selected motifs would not be co-occurring abundantly within an experiment (95th percentile) as well as be not too ubiquitous across ChIP-seq experiments (proximal: <20% and distal: <10%). The second threshold was chosen based upon the relative abundance of co-occurring motifs across experiments after applying the first threshold.

**Cell type specific **Motifs must co-occur in ChIP-seq peaks of experiments investigating the same cell type but across TFs (>3) and enriched with abundances above 15%. Motifs must co-occur in at least ten individual ChIP-seq experiments investigating the same cell type but different TF.

**TF specific **Motifs must co-occur with the same tar-get TF motif in at least three or more 'sister' experiments investigating other cell types. The motif must also have enrichment abundances above 20% in all experiments.

#### Identifying TFBS motifs in DNA sequence data from ChIP-seq

To scan for the relative occurrence of TFBS motif PWMs within ChIP-seq peaks, we used the TFBS motif identification software, FIMO, for Find Individual Motif Occurrences. FIMO is found as a part of the MEME suite of motif analysis algorithms and requires PWM(s) of the queried TFBS motif for scanning and identifying TFBS motifs in the sequences provided. When comparing two motifs, FIMO assigns a log-likelihood ratio score to each sequence position matched and the scores are converted into a *p*-value representing the probability of a true match. A corresponding q-value is generated using a bootstrap method to determine the mini-mal false discovery rate at which the *p*-value will be deemed significant [54].

#### Evaluating motif similarity

The varying degrees of similarity between motifs found in the compiled list of motifs were determined using the motif similarity comparison software, TOMTOM. TOMTOM creates ungapped alignments between queried motifs against a database of PWMs and determines a *p*-value describing the significance of the match against the rest of the motifs in the database. [55]. For this experiment all com-piled motifs were used as the reference database.

Motifs with *p*-values scores less than 0.05, when compared with the target TF's motif(s) were considered to be false positives.

## Competing interests

The authors declare that they have no competing interests.

## Authors' contributions

Experimental design by CX and PL and data analysis by GCL with assistance from CX. Manuscript written by GCL with input from CX and PL. All authors read and approved the final manuscript.

## Supplementary Material

Additional file 1 **Proximal co-occurring motifs**. List of co-occurring motifs identified in proximity (in the same ChIP-seq peak) with a target TF canonical motif with accompanying parameters. (.csv) Click here for file

Additional file 2 **Distal co-occurring motifs**. List of co-occurring motifs identified distally (in different ChIP-seq peak) with a target TF canonical motif with accompanying parameters. (.csv) Click here for file

Additional file 3 **Cell type specific motifs**. List of cell type specific co-occurring motifs. (.csv) Click here for file

Additional file 4 **TF specific motifs**. List of TF specific co-occurring motifs. (.csv) Click here for file

## Declaration

Publication of this article was supported by a grant to PL from the National University of Singapore (Grant R-069-000-047).

This article has been published as part of *BMC Genomics *Volume 14 Supplement 5, 2013: Twelfth International Conference on Bioinformatics (InCoB2013): Computational biology. The full contents of the supplement are available online at http://www.biomedcentral.com/bmcgenomics/supplements/14/S5.
